# Prevalence of methicillin-resistant *Staphylococcus aureus* (MRSA) infection and the molecular characteristics of MRSA bacteraemia over a two-year period in a tertiary teaching hospital in Malaysia

**DOI:** 10.1186/s12879-017-2384-y

**Published:** 2017-04-13

**Authors:** Pik San Sit, Cindy Shuan Ju Teh, Nuryana Idris, I-Ching Sam, Sharifah Faridah Syed Omar, Helmi Sulaiman, Kwai Lin Thong, Adeeba Kamarulzaman, Sasheela Ponnampalavanar

**Affiliations:** 1 Department of Medical Microbiology, Faculty of Medicine, University of Malaya, 50603 Kuala Lumpur, Malaysia; 2 Department of Medicine, Faculty of Medicine, University of Malaya, 50603 Kuala Lumpur, Malaysia; 3 Institute of Biological Sciences, Faculty of Science, University of Malaya, 50603 Kuala Lumpur, Malaysia

**Keywords:** Methicillin-resistant *Staphylococcus aureus* (MRSA), SCC*mec* types, MLST, PFGE, MRSA Bacteraemia

## Abstract

**Background:**

Methicillin-resistant *Staphylococcus aureus* (MRSA) is an established pathogen that causes hospital- and community-acquired infections worldwide. The prevalence rate of MRSA infections were reported to be the highest in Asia. As there is limited epidemiological study being done in Malaysia, this study aimed to determine the prevalence of MRSA infection and the molecular characteristics of MRSA bacteraemia.

**Methods:**

Two hundred and nine MRSA strains from year 2011 to 2012 were collected from a tertiary teaching hospital in Malaysia. The strains were characterized by antimicrobial susceptibility testing, staphylococcal cassette chromosome *mec* (SCC*mec*) typing, detection of Panton-Valentine leukocidin (PVL) gene, multilocus sequence typing (MLST) and pulsed-field gel electrophoresis (PFGE). Patient’s demographic and clinical data were collected and correlated with molecular data by statistical analysis.

**Results:**

Male gender and patient >50 years of age (*p* < 0.0001) were significantly associated with the increased risk of MRSA acquisition. Fifty-nine percent of MRSA strains were HA-MRSA that carried SCC*mec* type II, III, IV and V while 31% were CA-MRSA strains with SCC*mec* III, IV and V. The prevalence of PVL gene among 2011 MRSA strains was 5.3% and no PVL gene was detected in 2012 MRSA strains. All of the strains were sensitive to vancomycin. However, vancomycin MIC creep phenomenon was demonstrated by the increased number of MRSA strains with MIC ≥1.5 μg/mL (*p* = 0.008) between 2011 and 2012. Skin disease (*p* = 0.034) and SCC*mec* type III (*p* = 0.0001) were found to be significantly associated with high vancomycin MIC. Forty-four percent of MRSA strains from blood, were further subtyped by MLST and PFGE. Most of the bacteraemia cases were primary bacteraemia and the common comorbidities were diabetes, hypertension and chronic kidney disease. The predominant pulsotype was pulsotype C exhibited by SCC*mec* III-ST239. This is a first study in Malaysia that reported the occurrence of MRSA clones such as SCC*mec* V-ST5, untypeable-ST508, SCC*mec* IV-ST1 and SCC*mec* IV-ST1137.

**Conclusions:**

SCC*mec* type III remained predominant among the MRSA strains in this hospital. The occurrence of SCC*mec* IV and V among hospital strains and the presence of SCC*mec* III in CA-MRSA strains are increasing. MRSA strains causing bacteraemia over the two-year study period were found to be genetically diverse.

## Background

Methicillin-resistant *Staphylococcus aureus* (MRSA) is widely recognized as one of the pathogens causing hospital- and community- acquired infections. MRSA is highly prevalent in hospitals worldwide in which high rates (>50%) were reported in Asia, Malta, North and South America [1]. The prevalence of MRSA in Malaysia ranged from 17% in 1986 [2] to 44.1% in 2007 [3]. MRSA is evolved from methicillin-susceptible *S. aureus* through the acquisition of Staphylococcal cassette chromosome *mec* (SCC*mec*) which carries *mec*A gene. *mec*A gene encodes the penicillin-binding protein (PBP2a) which confers resistance to all β-lactam antibiotics [4].

Hospital-acquired (HA)-MRSA strains cause nosocomial infections and are associated with SCC*mec* type I, II or III. In the 1990s, the epidemiology of MRSA infections has changed due to the emergence of community-acquired (CA)-MRSA strains. CA-MRSA strains cause skin and soft tissue infections (SSTIs), sepsis, osteomyelitis, necrotizing pneumonia and fasciitis and pyomyositis in children, soldiers, professional football players or incarcerated populations. They often carry Panton-Valentine leukocidin (PVL) genes and SCC*mec* IV or V [4, 5]. To date, eleven different SCC*mec* types (I-XI) have been defined [6]. However, only SCC*mec* type I-V are globally distributed while others are uncommon and may exist as local strains in their original country [7].

There are various discriminative methods to type MRSA and to determine its molecular epidemiology. These methods include SCC*mec* typing, pulsed-field gel electrophoresis (PFGE) and multilocus sequence typing (MLST) [1]. In brief, SCC*mec* polymerase chain reaction (PCR) typing allows the presumptive assignment of all SCC*mec* types to MRSA strains which differentiates between HA-MRSA and CA-MRSA [8]. PFGE is based on DNA fingerprints generated by rare restriction endonuclease digestion and is useful for investigating nosocomial outbreaks as well as to identify MRSA strains that may cause major outbreaks. MLST is a highly discriminatory method used for the investigation of the molecular evolution of MRSA. It is based on the sequences of 450-bp internal fragments of 7 housekeeping genes amplified by PCR. The sequences of the genes are compared to those in MLST website (http://www.mlst.net ) which results in an allelic profile or sequence type (ST). By using the eBURST software (http://eburst.mlst.net/v3/mlst_datasets/), clonal complex (CC) can be defined based upon the STs, in which the evolutionary events can be analyzed [8, 9].

The most prevalent CCs reported worldwide are CC8 (ST239), CC5 (ST5) and CC22 (ST22) [1]. Numerous genetic linkages of CA-MRSA have been reported to be globally distributed. These include ST30-IV, ST8-IV, ST1-IV, ST59-V and ST80-IV [10]. Previous studies done by Lim et al. [11, 12] on 2003 and 2008 MRSA isolates in this same tertiary teaching hospital and on 2008 to 2010 MRSA isolates in a tertiary hospital in Terengganu had reported SCC*mec* type III and MLST type ST239 to be the predominant clone. Similar findings were also reported by Ghaznavi-Rad et al. [13], where 90% of MRSA infections in another tertiary hospital belonged to MLST type ST239. CA-MRSA strains in Malaysian hospitals were reported to belong to *SCCmec* type IV-MLST type ST30 based on study done by Ahmad et al. [3] on 2006 and 2008 MRSA strains from nine Malaysian hospitals.

Nosocomial bacteraemia has been reported to be common in hospitals worldwide leading to high mortality rate [14] and in Malaysia, 21% cases of bacteraemia were reported to be caused by MRSA [15]. International MRSA clones such as ST22-SCC*mec* IV, ST239-SCC*mec* III, ST5-SCC*mec* II [16] ST30-SCC*mec* IV and ST1-SCC*mec* IV [17] were reported to be spreading around the world, disseminating between and within the countries. HA-MRSA strains had also been found circulating in the community and CA-MRSA strains were reported to cause the outbreak of HA infections [18]. These epidemiological changes in MRSA have posed great threat to the public health. However, limited epidemiological studies have been done in Malaysian hospitals to monitor the trends in MRSA infections. The importance of continuous surveillance programs and molecular epidemiological studies; apart from proper infection control practices and antibiotic stewardship in designing effective and rational pathogen control strategies in hospitals were highlighted in an example of decreased rate of MRSA infections in Japan and Taiwan from 71.6% in 2001 to 41% in 2011 and 68.8% in 2000 to 55.9% in 2010, respectively [19] Therefore, the objectives of the study were to characterize the MRSA strains from year to 2011 to 2012 and to determine the characteristics of MRSA bacteraemia in the tertiary teaching hospital by molecular typing such as SCC*mec* and PVL PCR typing, MLST and PFGE.

## Methods

### Setting

UMMC is a 980-bed referral and teaching hospital in Malaysia consisting of intensive care units, paediatric, orthopaedic, surgical, medical, obstetrics and gynaecology and psychiatry wards and clinics.

### Bacterial strains

All adult patients (>16 years old) who fulfilled the criteria for MRSA infection were included in this study. This study was approved by Medical Ethic Committee of University Malaya Medical Centre (UMMC) on 7th June 2014 (MEC ID: 20145-168). A total of 209 non-duplicate MRSA clinical strains were collected retrospectively from University Malaya Medical Centre (UMMC). The strains were previously isolated from sterile sites such as cerebrospinal fluid (CSF), synovial fluid, tissue, bone, pus and blood in January 2011 to December 2012. Only strains from pus that was clinically significant and obtained in an aseptic manner either in operation theatre (OT) or procedure room was included in this study. The identities of the strains were confirmed with PCR targeting *fem*A gene and identified as MRSA using standard microbiological methods such as tube coagulase test, DNase and cefoxitin disk (FOX) screen test.

These strains were characterized using antimicrobial susceptibility testing, SCC*mec* typing and PCR targeting PVL gene. Ninety-one selected strains isolated from blood were further subtyped by PFGE and MLST.

### Antimicrobial susceptibility testing

Vancomycin minimum inhibitory concentration (MIC) results based on E-test were collected from the hospital’s microbiology diagnostic laboratory. The interpretation was done according to Clinical and Laboratory Standards Institute (CLSI) guidelines.

### SCC*mec* typing and PCR-based assays for PVL gene

Crude genomic DNA was extracted by using simple boiling method and the supernatant was used as DNA template for PCR analysis. The cycling condition and primers as described by Milheirico et al. [20] were used to detect SCC*mec* types. For further subgrouping SCC*mec* type IV strains, the cycling condition as described by Milheirico et al. [21] and specific primers 4a1, 4a2, 4b1, 4b2, 4c1, 4c2, 4d1 and 4d2 as described by Zhang et al. [22] were used. The following strains: 85/2082 (SCC*mec* type III), JCSC2172 (SCC*mec* type IVb), JCSC4744 (SCC*mec* type IVa), JCSC4469 (SCC*mec* type IVd), MR108 (SCC*mec* type IVc), N315 (SCC*mec* type II), NCTC10442 (SCC*mec* type I) and WIS (SCC*mec* type V) were kindly provided by K.L. Thong, University of Malaya and used as positive control strains to optimize the multiplex PCR assay. The detection of PVL gene was performed as described by Holmes et al. [23] with slight modification. Briefly, the amplification was performed in a final volume of 25 μl containing 2 μl of DNA template, 1X PCR buffer, 1.5 mM MgCI_2_, 0.2 mM dNTPs, 0.5 μl of Taq polymerase (Promega Corporation, USA) and 0.2 μl of primers *luk-PV*-1 and *luk-PV*-2 (Integrated DNA Technologies, USA). The cycling conditions were as described and the PCR products were analyzed by electrophoresis in a 1% agarose gel.

### MLST and PFGE

MLST was performed as previously described by Enright et al. [24]. The sequence types (STs) were assigned by comparing the sequences of each locus to those in the *S. aureus* MLST database (http://saureus.mlst.net) and clonal complexes were defined by eBURST program.

PFGE was performed according to the Centers for Disease Control and Prevention (CDC) PulseNet protocol [25] with slight modification. In brief, a single colony of bacteria was streaked onto TSA (BD Difco™) and incubated at 37 °C overnight. An aliquot of 100 μl of bacterial cell suspension (containing bacterial culture in cell suspension buffer) was transferred to a microcentrifuge tube and added with lysostaphin (1 mg/mL) and lysozyme (10 mg/mL) (Sigma-Aldrich, USA). Following incubation at 37 °C, proteinase K (20 mg/mL) (Promega Corporation, USA) and 1% seakem gold agarose (Lonza, USA) were added into the suspension, mixed and allowed to solidify in a plug mold. The plug was transferred into cell lysis buffer (CLB), incubated at 54 °C for 3 h and washed with sterile distilled water and TE buffer. A slice of plug was cut and digested with *Sma*I (Promega Corporation, USA) followed by separation on CHEF MAPPER in 0.5X TBE at 14 °C for 22 h with pulse time of 5 – 60 s. The *Salmonella ser*. Branderup isolate H9812 was used as the reference strain. The gel was stained in Gel Red dye (Biotium, USA) and visualized under BioRad GelDoc XR. BioNumerics 6.5 Software package was used for cluster analyses of PFGE profiles based on the unweighted pair group method with arithmetic averages (UPGMA) with a tolerance of 1.5% and optimization of 1%. The PFGE profile was assigned an arbitrary designation and the Dice coefficient of similarity, *F* defined the differences [11].

### Clinical data collection

Patient’s demographic and clinical data such as age, gender, diagnosis, comorbidities, site of infection, healthcare- associated risk factors were collected from the hospital’s medical record unit. Based on the CDC definitions, HA-MRSA is defined as positive culture obtained more than 48 h after hospital admission, or history of previous hospitalization or medical procedures. CA-MRSA refers to cases with positive culture obtained less than 48 h of admission without healthcare-associated risk factors [18, 26]. In this study, MRSA infection were defined as HA- or CA- MRSA based upon the data collected from patient’s clinical notes and from the Infection Control Department’s database on multidrug resistant organisms (MDROs). By using these two sources, the risk factors for HA- and CA- MRSA infections were identified with reasonable accuracy.

### Statistical analysis

Categorical data were compared using Fisher exact test. All reported *p* values are two-tailed and analyses were performed using GraphPad software (https://www.graphpad.com/quickcalcs/catMenu/). Variables with *p* < 0.05 were considered to be statistically significant.

## Results

### Distribution of MRSA strains

In this study, 209 MRSA strains from year 2011 to 2012 were collected from various clinical specimens including tissues (*n* = 79; 37.8%), blood (*n* = 91; 43.5%), pus, slough and abscess (*n* = 19; 9.1%), cerebrospinal fluid (*n* = 3; 1.4%), bone (*n* = 11; 5.3%), pericardial fluid (*n* = 1; 0.5%), bullae fluid (*n* = 1; 0.5%) and synovial fluid (*n* = 4; 1.9%). The median age was 58 years old ranging from 16 to 92 years old and most cases belonged to the age group of 51–92 years. MRSA infections were significantly decreased in the age group of ≤50 years old (41.1% in 2011 vs 23.7% in 2012) (*p* = 0.011) while an increase was seen in the age group of >50 years old (54.7% in 2011 vs 75.4% in 2012) (*p* = 0.002). A total of 132 (63.2%) specimens were collected from male, 74 (35.4%) were from female and 3 specimens were unknown. Age > 50 years old and male gender (*p* < 0.0001) were the significant risk factors in the acquisition of MRSA.

Fifty-nine percent (123 of 209) of MRSA strains were HA-MRSA, 31% (65 of 209) were of CA-MRSA and the remaining MRSA strains were not known. Some of the MRSA strains had incomplete clinical data such as unknown age, gender and MRSA types because the patient’s medical records could not be retrieved from the archived records. HA- and CA- MRSA infections were significantly increased and decreased from year 2011 to 2012 (*p* = 0.001) respectively (Table 1).

**Table 1:** Comparisons between the patients’ demographics, phenotypic and genotypic characteristics of HA-MRSA and CA-MRSA in 2011 and 2012

	2011	2012	*P* value	Total	*P* value
	*N* = 95 (%)	*N* = 114 (%)		*N* = 209 (%)	
Age					
≤ 50 years old	39 (41.1)	27 (23.7)	**0.011**	66 (31.6)	**< 0.0001**
> 50 years old	52 (54.7)	86 (75.4)	**0.002**	138 (66)	
Not Known	4 (4.2)	1 (0.9)		5 (2.4)	
Gender					
Female	30 (31.6)	44 (38.6)	0.312	74 (35.4)	**< 0.0001**
Male	62 (65.3)	70 (61.4)	0.666	132 (63.2)	
Not Known	3 (3.2)	0 (0)		3 (1.4)	
Source					
Blood	40 (42.1)	51 (44.7)	0.780	91 (43.5)	
Tissue	37 (38.9)	42 (36.8)	0.776	79 (37.8)	
CSF	3 (3.2)	0 (0)	0.092	3 (1.4)	
Pus, slough & Abscess	8 (8.4)	11 (9.6)	0.813	19 (9.1)	
Pericardial fluid	1 (1.1)	0 (0)	0.455	1 (0.5)	
Bullae fluid	0 (0)	1 (0.9)	1.000	1 (0.5)	
Synovial fluid	2 (2.1)	2 (1.8)	1.000	4 (1.9)	
Bone	4 (4.2)	7 (6.1)	0.758	11 (5.3)	
SCC*mec* types					
SCC*mec* I	0 (0)	0 (0)		0 (0)	
SCC*mec* II	2 (2.1)	0 (0)	0.205	2 (0.9)	
SCC*mec* III	61 (64.2)	78 (68.4)	0.558	139 (66.5)	
SCC*mec* IV					
SCC*mec* IVa	5 (5.3)	4 (3.5)	0.735	9 (4.3)	
SCC*mec* IVb	5 (5.3)	0 (0)	**0.018**	5 (2.4)	
SCC*mec* IVc	0 (0)	0 (0)		0 (0)	
SCC*mec* IVd	0 (0)	0 (0)		0 (0)	
Novel subtypes	15 (15.8)	30 (26.3)	0.090	45 (21.5)	
SCC*mec* V	6 (6.3)	1 (0.9)	**0.048**	7 (3.3)	
Untypeable	1 (1.1)	1 (0.9)	1.000	2 (0.9)	
Type of MRSA					
HA-MRSA	44 (46.3)	79 (69.3)	**0.001**	123 (58.9)	
SCC*mec* II	2 (4.5)	0 (0)	0.126	2 (1.6)	
SCC*mec* III	35 (79.5)	56 (70.9)	0.392	91 (73.9)	
SCC*mec* IV					
SCC*mec* IVa	1 (2.3)	3 (3.8)	1.000	4 (3.3)	
SCC*mec* IVb	3 (6.8)	0 (0)	**0.044**	3 (2.4)	
Novel subtypes	3 (6.8)	19 (24.1)	**0.025**	22 (17.9)	
SCC*mec* V	0 (0)	1 (1.3)	1.000	1 (0.8)	
CA-MRSA	41 (43.2)	24 (21.1)	**0.001**	65 (31.1)	
SCC*mec* III	23 (56.1)	13 (54.2)	1.000	36 (55.4)	
SCC*mec* IV					
SCC*mec* IVa	4 (9.8)	1 (4.2)	0.644	5 (7.7)	
SCC*mec* IVb	2 (4.9)	0 (0)	0.527	2 (3.1)	
Novel subtypes	7 (17.1)	10 (41.7)	**0.042**	17 (26.2)	
SCC*mec* V	4 (9.8)	0 (0)	0.288	4 (6.2)	
Untypeable	1 (2.4)	0 (0)	1.000	1 (1.5)	
Not Known	10 (10.5)	11 (9.6)		21 (10)	
SCC*mec* III	3 (30)	9 (81.8)		12 (57.1)	
SCC*mec* IV					
Novel subtypes	5 (50)	1 (9.1)		6 (28.6)	
SCC*mec* V	2 (20)	0 (0)		2 (9.5)	
Untypeable	0 (0)	1 (9.1)		1 (4.8)	
PVL gene	5 (5.3)	0 (0)	**0.018**	5 (2.4)	
Vancomycin MIC					
< 1.5 μg/mL	58 (61.1)	48 (42.1)	**0.008**	106 (50.7)	
≥ 1.5 μg/mL	37 (38.9)	66 (57.9)		103 (49.3)	

*CSF* Cerebrospinal fluid

Fisher’s exact test done for categorical variables, *p* value < 0.05 was considered to be statistical significant ﻿and are indicated by bold text﻿

### Antimicrobial susceptibility testing

All the MRSA strains were sensitive to vancomycin with the vancomycin MICs ranged from 0.38 – 2 μg/mL. One hundred and six (50.7%) and 103 (49.3%) out of 209 strains had vancomycin MIC <1.5 μg/mL and ≥1.5 μg/mL, respectively. A significant decreased was observed in the number of strains with MIC <1.5 μg/mL (61.1% in 2011 vs 42.1% in 2012) and an increase was seen in the number of strains with MIC ≥1.5 μg/mL (38.9% in 2011 vs 57.9% in 2012) (*p* = 0.008). Comparisons of patient demographics, clinical features and the genotypes between the low (<1.5 μg/mL) and high (≥ 1.5 μg/mL) vancomycin MIC groups were shown in Table 2. There were no significant differences in terms of age, gender and clinical diagnosis. However, skin disease (*p* = 0.034) and SCC*mec* type III (*p* = 0.0001) were found to be strongly associated with elevated MIC (≥ 1.5 μg/mL), while SCC*mec* type IV was associated with low vancomycin MIC (*p* = 0.0001) (Table 2).

**Table 2:** Comparison between the vancomycin MICs with patient’s demographics, clinical diagnosis and genotypic characteristics of MRSA in 2011 and 2012

	Vancomycin MIC						
	< 1.5 μg/mL			≥ 1.5 μg/mL			*P* value
	2011	2012	Total	2011	2012	Total	
	*N* = 58 (%)	*N* = 48 (%)	*N* = 106 (%)	*N* = 37 (%)	*N* = 66 (%)	*N* = 103 (%)	
Age							
≤ 50 years old	26 (44.8)	8 (16.7)	34 (32.1)	13 (35.1)	19 (28.8)	32 (31.1)	0.883
> 50 years old	29 (50)	40 (83.3)	69 (65.1)	23 (62.2)	46 (69.7)	69 (66.9)	0.884
Not Known	3 (5.2)	0 (0)	3 (2.8)	1 (2.7)	1 (1.5)	2 (1.9)	
Gender							
Female	18 (31)	22 (45.8)	40 (37.7)	12 (32.4)	22 (33.3)	34 (33)	0.563
Male	38 (65.5)	26 (54.2)	64 (60.4)	24 (64.9)	44 (66.7)	68 (66)	0.474
Not Known	2 (3.4)	0 (0)	2 (1.9)	1 (2.7)	0 (0)	1 (0.9)	
Clinical diagnosis							
Bacteraemia	25 (43.1)	24 (50)	49 (46.2)	15 (40.5)	27 (40.9)	42 (40.8)	0.486
Skin and soft tissue infections	26 (44.8)	20 (41.7)	46 (43.4)	20 (54.1)	31 46.9)	51 (49.5)	0.407
Osteomyelitis or septic arthritis	3 (5.2)	3 (6.3)	6 (5.7)	2 (5.4)	7 (10.6)	9 (8.7)	0.432
Meningitis	3 (5.2)	0 (0)	3 (2.8)	0 (0)	1 (1.5)	1 (0.9)	0.622
Pericarditis	1 (1.7)	1 (2.1)	2 (1.9)	0 (0)	0 (0)	0 (0)	0.498
Co-morbidities							
Diabetes mellitus	12 (20.7)	27 (56.3)	39 (36.8)	12 (32.4)	25 (37.9)	37 (35.9)	1.000
Hypoglycaemia	1 (1.7)	0 (0)	1 (0.9)	0 (0)	0 (0)	0 (0)	1.000
Hypertension	12 (20.7)	24 (50)	36 (33.9)	11 (29.7)	23 (34.8)	34 (33)	1.000
Obesity	0 (0)	1 (2.1)	1 (0.9)	0 (0)	0 (0)	0 (0)	1.000
Chronic kidney disease and UTI	9 (15.5)	19 (39.6)	28 (26.4)	5 (13.5)	25 (37.9)	30 (29.1)	0.758
Cancer	8 (13.8)	6 (12.5)	14 (13.2)	3 (8.1)	2 (3)	5 (4.9)	0.052
Head injury	3 (5.2)	10 (20.8)	13 (12.3)	6 (16.2)	10 (15.2)	16 (15.5)	0.552
Liver disease	1 (1.7)	5 (10.4)	6 (5.7)	0 (0)	2 (3)	2 (1.9)	0.280
Respiratory disease	7 (12.1)	9 (18.8)	16 (15.1)	3 (8.1)	10 (15.2)	13 (12.6)	0.691
Cardiovascular disease	6 (10.3)	11 (22.9)	17 (16)	2 (5.4)	8 (12.1)	10 (9.7)	0.217
Gastrointestinal disease	2 (3.4)	3 (6.3)	5 (4.7)	0 (0)	2 (3)	2 (1.9)	0.446
Autoimmune disease	1 (1.7)	0 (0)	1 (0.9)	0 (0)	0 (0)	0 (0)	1.000
Bone and joint disorder	3 (5.2)	4 (8.3)	7 (6.6)	0 (0)	6 (9.1)	6 (5.8)	1.000
Endocrine disorder	0 (0)	2 (4.2)	2 (1.9)	0 (0)	1 (1.5)	1 (0.9)	1.000
Blood disorder	1 (1.7)	1 (2.1)	2 (1.9)	0 (0)	2 (3)	2 (1.9)	1.000
CMV	0 (0)	1 (2.1)	1 (0.9)	0 (0)	0 (0)	0 (0)	1.000
Skin disease	0 (0)	1 (2.1)	1 (0.9)	4 (10.8)	3 (4.5)	7 (6.8)	**0.034**
None	3 (5.2)	4 (8.3)	7 (6.6)	0 (0)	4 (6.1)	4 (3.9)	0.538
Not Known	24 (41.4)	2 (4.2)	26 (24.5)	12 (32.4)	8 (12.1)	20 (19.4)	0.407
SCC*mec* types							
SCC*mec* II	0 (0)	0 (0)	0 (0)	2 (5.4)	0 (0)	2 (1.9)	0.242
SCC*mec* III	32 (55.2)	21 (43.8)	53 (50)	29 (78.4)	57 (86.4)	86 (83.5)	**0.0001**
SCC*mec* IV							
SCC*mec* IVa	4 (6.9)	3 (6.3)	7 (6.6)	1 (2.7)	1 (1.5)	2 (1.9)	0.171
SCC*mec* IVb	5 (8.6)	0 (0)	5 (4.7)	0 (0)	0 (0)	0 (0)	0.060
Novel subtypes	13 (22.4)	23 (47.9)	36 (33.9)	2 (5.4)	7 (10.6)	9 (8.7)	**0.0001**
SCC*mec* V	3 (5.2)	1 (2.1)	4 (3.8)	3 (8.1)	0 (0)	3 (2.9)	1.000
Untypeable	1 (1.7)	0 (0)	1 (0.9)	0 (0)	1 (1.5)	1 (0.9)	1.000

*UTI* = Urinary Tract Infection, *CMV* = Cytomegalovirus

Head injury includes: basal ganglia bleed, subdural hematoma and stroke. Liver disease includes: alcoholic liver disease, liver cirrhosis. Respiratory disease includes: pneumonia, pulmonary embolism, chronic obstructive pulmonary disease, acute respiratory distress syndrome. Cardiovascular disease includes: mitral valve regurgitation, ischaemic heart disease, acute coronary syndrome, congestive cardiac failure. Gastrointestinal disease includes: acute gastroenteritis, Crohn’s disease, perforated diverticular disease, intestinal obstruction. Autoimmune disease includes: Systemic lupus erythematosus. Endocrine disorder includes: thyroid disease, primary hypothyroidism. Blood disorder includes: myelofibrosis, anaemia. Skin disease includes: Stevens Johnson Syndrome, Bullous pemphigoid, exfoliative dermatitis and pemphigus vulgaris

Fisher’s exact test done for categorical variables, *p* value < 0.05 was considered to be statistical significant and are indicated by bold text

### SCC*mec* types and the presence of PVL gene

For year 2011 MRSA strains, the predominant SCC*mec* type was SCC*mec* type III (*n* = 61; 64.2%). A total of 25 MRSA strains were SCC*mec* type IV and further subtyped into SCC*mec* type IVa (*n* = 5; 5.3%) and SCC*mec* type IVb (*n* = 5; 5.3%). Fifteen MRSA strains could not be subtyped and were known as novel SCC*mec* type IV subtypes. There were two and six strains typed as SCC*mec* type II and SCC*mec* type V, respectively. For 2012 MRSA strains, three SCC*mec* types were observed: SCC*mec* type III (*n* = 78; 68.4%), SCC*mec* type IV (*n* = 34; 29.8%) and SCC*mec* type V (*n* = 1; 0.9%). The SCC*mec* type IV strains were further subtyped as SCC*mec* type IVa (*n* = 4; 3.5%) and novel type IV SCC*mec* subtypes (*n* = 30; 26.3%). There were two untypeable MRSA strains which harbour *mec*A gene and no SCC*mec* type I was found in both years. PVL gene was present in five MRSA strains from 2011 but none was reported in 2012 MRSA strains. All PVL-positive strains were CA-MRSA strains isolated from pus, tissue and abscess and carried SCC*mec* type IV and V. There was no significant difference in the number of strains carrying SCC*mec* types II and III. However, a significant decreased in the numbers of SCC*mec* types IVb (*p* = 0.018) and V (*p* = 0.048) from 2011 to 2012 was observed.

### Genotyping by PFGE

**Fig. 1:**
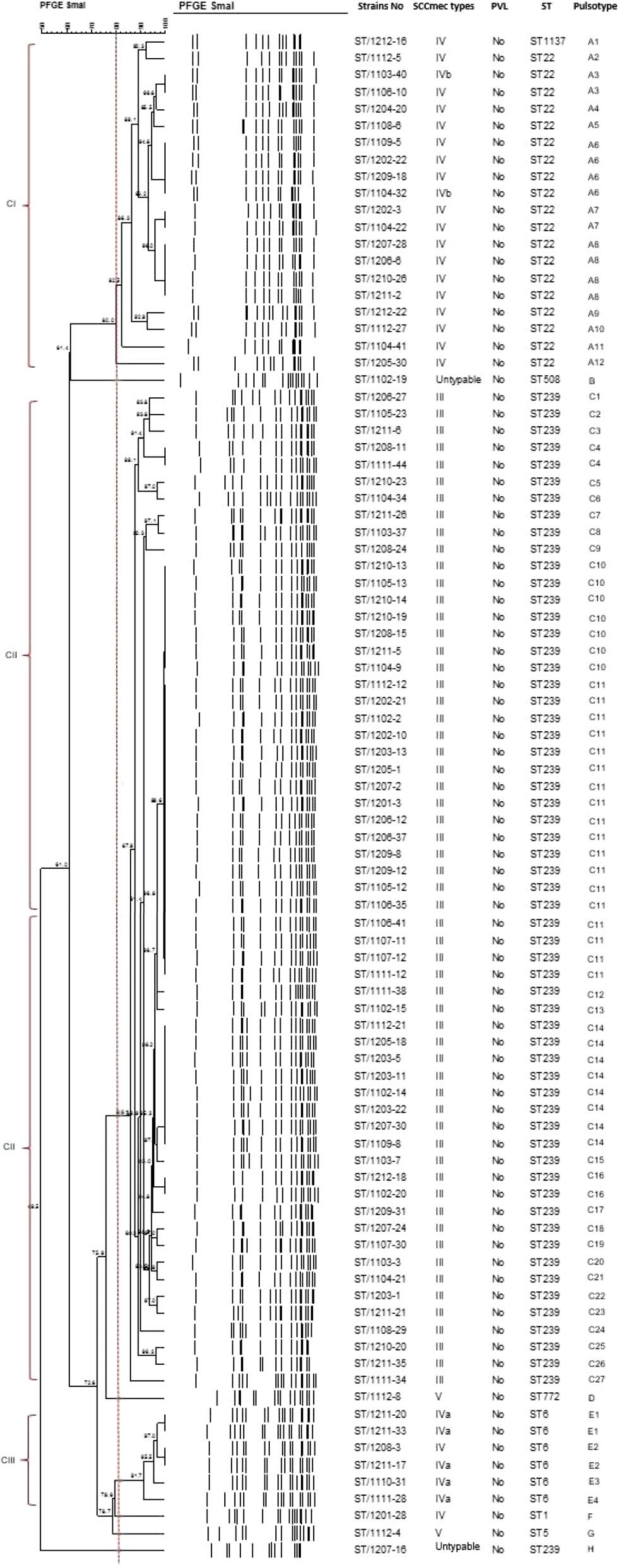
Dendrogram of MRSA PFGE patterns generated by UPGMA clustering method using Dice coefficient. The dotted line indicates an arbitrary 80% similarity demarcation

PFGE of *Sma*I-digested chromosomal DNA of 91 MRSA strains generated 8 pulsotypes (A – H) each with 13 – 20 bands, with a Dice coefficient, *F* ranging from 0.5 to 1.0. Based on 80% similarity, three clusters (Cluster I to Cluster III) and five singletons were observed. The majority of the strains (64.8%) were clustered in Cluster II, followed by Cluster I which contained 20 strains (21.9%) and Cluster III which contained 9 strain (6.6%). Pulsotype C in Cluster II was the predominant pulsotype seen with multiple subtypes (C1- C27) and followed by pulsotype A in Cluster I with subtypes (A1-A12). Pulsotype E in Cluster III was less common with fewer subtypes (E1-E4) and pulsotypes B, D, F, G and H had no subtypes. Most of the MRSA strains belonged to subtype C11, which was the top major subtype and they were indistinguisable or clonally related. The presence of the clonally or closely related MRSA strains despite being isolated from different wards and different years may suggest the persistence of this clone in this hospital and there was transmission of this clone between wards (Fig. 1).

### Determination of clonal relationships by MLST

**Fig. 2:**
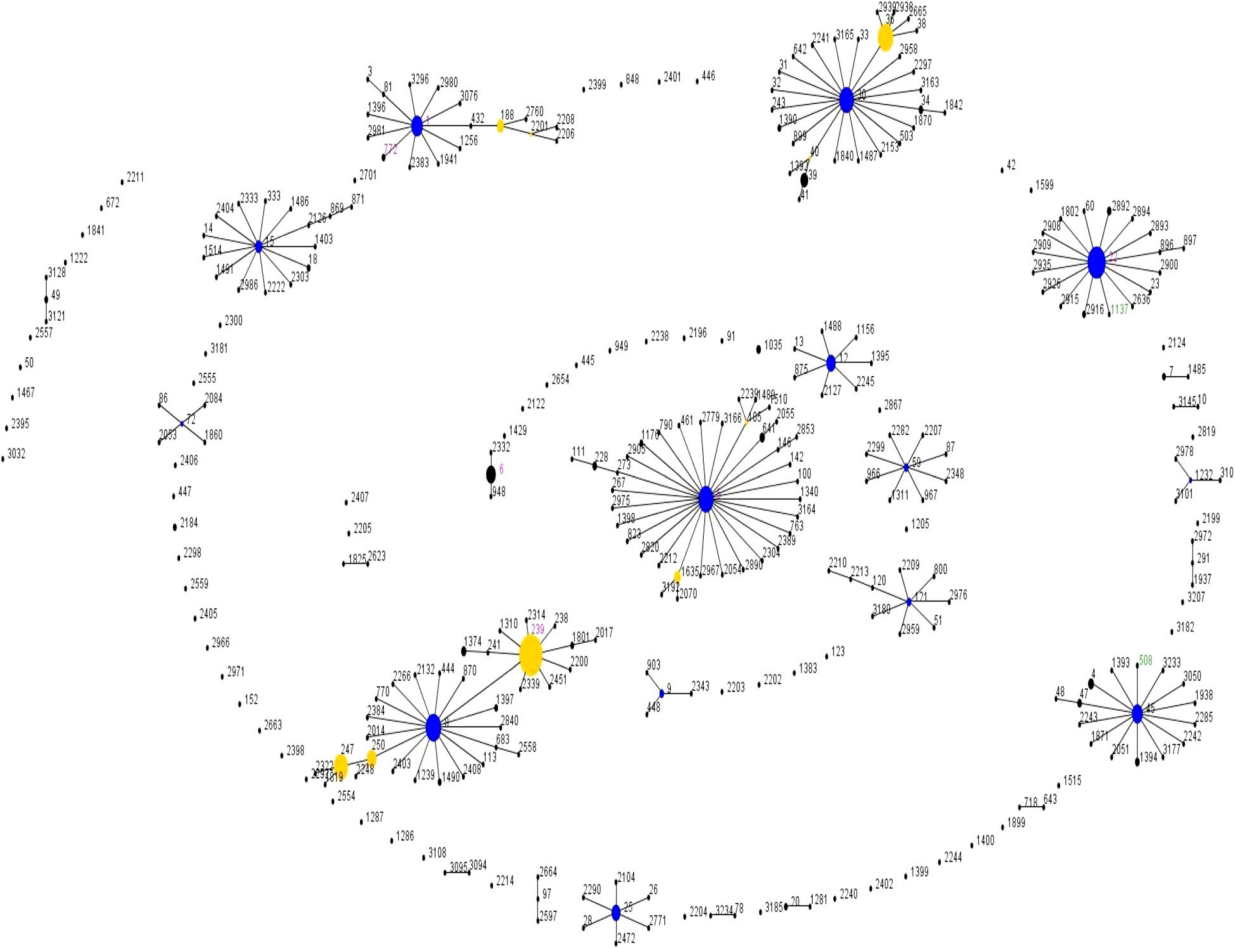
Population snapshot of MRSA strains from blood source in the MLST database. Individual sequence type (ST) is highlighted in black (STs in the database), green (STs in this study) and pink (STs in both the database and in this study) and is represented by each circle. The size of the circle indicates the frequency of a particular ST and the colour of the circle represents founders or subgroup founders. Blue circles are ‘founders’ [defined as ST with many single locus variants (SLVs) and is prevalent within a CC], whereas yellow circle are ‘subgroup founders’. The most prevalent ST in this study was ST239, which is the subgroup founder within CC8

MLST analysis revealed eight dfferent sequence types (ST) which include ST239 (*n* = 61), ST22 (*n* = 19), ST508 (*n* = 1), ST772 (*n* = 1), ST6 (*n* = 6), ST5 (*n* = 1), ST1 (*n* = 1) and ST1137 (*n* = 1). ST6, ST22 and ST239 were observed in MRSA strains of both years, whereas ST5, ST508 and ST772 were present among the 2011 MRSA strains and ST1 and ST1137 were found in 2012 MRSA strains. SCC*mec* type III belonged to ST239, SCC*mec* type IV belonged to ST1, ST6, ST22 and ST1137 while SCC*mec* type V belonged to ST5 and ST772. Strains with untypeable SCC*mec* were represented by two STs (239 and 508). MRSA strains were clustered into six clonal complexes (CC) based on the similarity between STs in six of seven loci. ST239 was assigned as the putative ancestral genotype of a subgroup within CC8. ST1, ST5, ST6 and ST22 were identified as the ancestral genotypes of their corresponding CCs. The newly discovered clones; ST508 and ST1137 were observed in this study and they belonged to CC45 and CC22, respectively (Fig. 2) (Table 3).

**Table 3:** Comparison between SCC*mec* types with the patient’s demographic, clinical diagnosis and molecular characteristics of MRSA strains isolated from blood

	SCC*mec* III	SCC*mec* IV	SCC*mec* V	Untypeable	Total
	*N* = 60 (%)	*N* = 27 (%)	*N* = 2 (%)	*N* = 2 (%)	*N* = 91 (%)
Age					
≤ 50 years old	12 (20)	3 (11.1)	0 (0)	0 (0)	15 (16.5)
> 50 years old	47 (78.3)	23 (85.2)	2 (100)	2 (100)	74 (81.3)
Not Known	1 (1.7)	1 (3.7)	0 (0)	0 (0)	2 (2.2)
Gender					
Female	30 (50)	10 (37)	0 (0)	0 (0)	40 (43.9)
Male	29 (48.3)	16 (59.3)	2 (100)	2 (100)	49 (53.8)
Not Known	1 (1.7)	1 (3.7)	0 (0)	0 (0)	2 (2.2)
Co-morbidities					
Diabetes mellitus	20 (33.3)	11 (40.7)	1 (50)	0 (0)	32 (35.2)
Hypertension	22 (36.7)	12 (44.4)	1 (50)	0 (0)	35 (38.5)
Obesity	0 (0)	1 (3.7)	0 (0)	0 (0)	1 (1.1)
Chronic kidney disease and UTI	24 (40)	14 (51.9)	0 (0)	0 (0)	38 (41.8)
Cancer	8 (13.3)	2 (7.4)	0 (0)	0 (0)	10 (10.9)
Head injury	11 (18.3)	4 (14.8)	0 (0)	1 (50)	16 (17.6)
Liver disease	4 (6.7)	2 (7.4)	0 (0)	0 (0)	6 (6.6)
Respiratory disease	12 (20)	3 (11.1)	1 (50)	0 (0)	16 (17.6)
Cardiovascular disease	4 (6.7)	8 (29.6)	2 (100)	0 (0)	14 (15.4)
Gastrointestinal disease	4 (6.7)	1 (3.7)	0 (0)	0 (0)	5 (5.5)
Autoimmune disease	1 (1.7)	0 (0)	0 (0)	0 (0)	1 (1.1)
Bone and joint disorder	1 (1.7)	2 (7.4)	0 (0)	0 (0)	3 (3.3)
Endocrine disorder	0 (0)	2 (7.4)	0 (0)	0 (0)	2 (2.2)
Blood disorder	2 (3.3)	0 (0)	0 (0)	0 (0)	2 (2.2)
CMV	1 (1.7)	0 (0)	0 (0)	0 (0)	1 (1.1)
Skin disease	7 (11.7)	0 (0)	0 (0)	0 (0)	7 (7.7)
None	1 (1.7)	0 (0)	0 (0)	0 (0)	1 (1.1)
Not known	7 (11.7)	5 (18.5)	0 (0)	1 (50)	13 (14.3)
No of comorbidities					
0	8 (13.3)	5 (18.5)	0 (0)	1 (50)	14 (15.4)
1	19 (31.7)	5 (18.5)	1 (50)	1 (50)	26 (28.6)
2	11 (18.3)	6 (22.2)	0 (0)	0 (0)	17 (18.7)
3	12 (20)	1 (3.7)	0 (0)	0 (0)	13 (14.3)
≥ 4	10 (16.7)	10 (37)	1 (50)	0 (0)	21 (23.1)
Source of bacteraemia					
Primary bacteraemia	38 (63.3)	19 (70.4)	2 (100)	2 (100)	61 (67)
Catheter-related	7 (11.7)	5 (18.5)	0 (0)	0 (0)	12 (13.2)
Skin and soft tissue infection	6 (10)	0 (0)	0 (0)	0 (0)	6 (6.6)
Surgical site infection	1 (1.7)	0 (0)	0 (0)	0 (0)	1 (1.1)
Pneumonia	5 (8.3)	2 (7.4)	0 (0)	0 (0)	7 (7.7)
Implant-related infection	0 (0)	1 (3.7)	0 (0)	0 (0)	1 (1.1)
More than one source	3 (5)	0 (0)	0 (0)	0 (0)	3 (3.3)
Type of MRSA					
HA-MRSA	36 (60)	15 (55.6)	0 (0)	0 (0)	51 (56)
CA-MRSA	15 (25)	9 (33.3)	2 (100)	1 (50)	27 (29.7)
Not Known	9 (15)	3 (11.1)	0 (0)	1 (50)	13 (14.3)
CC/ST					
*CC1*					
ST1	0 (0)	1 (3.7)	0 (0)	0 (0)	1 (1.1)
ST772	0 (0)	0 (0)	1 (50)	0 (0)	1 (1.1)
*CC5*					
ST5	0 (0)	0 (0)	1 (50)	0 (0)	1 (1.1)
*CC6*					
ST6	0 (0)	6 (22.2)	0 (0)	0 (0)	6 (6.6)
*CC8*					
ST239	60 (100)	0 (0)	0 (0)	1 (50)	61 (67)
*CC22*					
ST22	0 (0)	19 (70.4)	0 (0)	0 (0)	19 (20.9)
ST1137	0 (0)	1 (3.7)	0 (0)	0 (0)	1 (1.1)
*CC45*					
ST508	0 (0)	0 (0)	0 (0)	1 (50)	1 (1.1)
Vancomycin MIC					
< 1.5 μg/mL	22 (36.7)	25 (92.6)	1 (50)	1 (50)	49 (53.8)
≥ 1.5 μg/mL	38 (63.3)	2 (7.4)	1 (50)	1 (50)	42 (46.2)

*UTI* = Urinary Tract Infection, *CMV* = Cytomegalovirus

Head injury includes: basal ganglia bleed, subdural hematoma and stroke. Liver disease includes: alcoholic liver disease, liver cirrhosis. Respiratory disease includes: pneumonia, pulmonary embolism, chronic obstructive pulmonary disease, acute respiratory distress syndrome. Cardiovascular disease includes: mitral valve regurgitation, ischaemic heart disease, acute coronary syndrome, congestive cardiac failure. Gastrointestinal disease includes: acute gastroenteritis, Crohn’s disease, perforated diverticular disease, intestinal obstruction. Autoimmune disease includes: Systemic lupus erythematosus. Endocrine disorder includes: thyroid disease, primary hypothyroidism. Blood disorder includes: myelofibrosis, anaemia. Skin disease includes: Stevens Johnson Syndrome, Bullous pemphigoid, exfoliative dermatitis and pemphigus vulgaris

## Discussion

MRSA is known to be one of the most prominent pathogens that causes HA and CA- associated infections in Malaysian hospitals. In this study, 59% (123 of 209) of MRSA strains were HA-MRSA and 31% (65 of 209) were of CA-MRSA. Most of the HA-MRSA infections were caused by SCC*mec* type III (*n* = 91; 73.9%) strains. Although SCC*mec* IV and V have been reported to occur in the community [9], most patients seem to acquire these strains in this hospital based on the proportions of SCC*mec* IV (*n* = 29; 23.6%) and V (*n* = 1; 0.8%), indicating the invasion of these strains into hospitals and may replace the classical HA-MRSA strains due to their unique characteristics and faster growth patterns [27]. CA-MRSA infections in this hospital have decreased from 41 to 24 cases in 2011 and 2012, respectively. The high prevalence of SCC*mec* type III (*n* = 36; 55.4%) among the CA-MRSA strains might indicate that this MRSA hospital strain has spread to the community. As stated by Chen et al. [28] in a recent study in China, SCC*mec* III-ST239 is beginning to appear in CA-MRSA isolates. In addition, in the first international surveillance study on the CA-MRSA epidemiology in Asian countries, have revealed the spreading of HA-MRSA isolates to the community, given the presence of SCC*mec* types I, II and III in CA-MRSA isolates from Taiwan, Korea, Hong Kong, Philippines, Thailand and Vietnam [29]. Based on the distributions analysis in Table 1, MRSA infections were significantly correlated with male patients and between the ages of 51 and 92 years.

The existence of the vancomycin creep phenomenon was demonstrated by the increased percentages of MRSA strains with MIC ≥1.5 μg/mL over a 2-year period. In addition, skin disease and SCC*mec* type III were found to be significantly associated with high vancomycin MIC. This is possibly due to the previous exposure of MRSA strains to vancomycin which led to the changes in the characteristics of bacteria such as bacterial cell wall alterations, preventing vancomycin to reach its target site [30].

SCC*mec* type III was the predominant SCC*mec* type for year 2011 (*n* = 61; 64.2%) and 2012 (*n* = 78; 68.4%) which is consistent with previous reports in Malaysian hospitals done by Lim et al. [11] and Ghaznavi-Rad et al. [13]. SCC*mec* type III was also common in Asian countries such as Taiwan, Singapore, Thailand and Indonesia [19]. For year 2011 MRSA strains, 25 MRSA strains are SCC*mec* type IV and were further subtyped as SCC*mec* type IVa and IVb. Fifteen SCC*mec* type IV MRSA strains could not be further subtyped and might be the novel type IV SCC*mec* subtypes. SCC*mec* types II and V were also observed. For 2012 MRSA strains, other than SCC*mec* type III, SCC*mec* type IV and V were also observed. The SCC*mec* type IV strains are further subtyped as SCC*mec* type IVa and novel type IV SCC*mec* subtypes. There were untypeable MRSA strains which harbours *mec*A gene detected in each year. These strains may indicate new or variant SCC*mec* types which should be further analysed [31]. The prevalence of PVL gene among 2011 MRSA strains was 5.3% and most of them caused skin and soft tissue infections. No PVL gene was detected in year 2012 strains.

MRSA bacteraemia is common in hospitals worldwide, including Malaysia and is associated with high mortality rate and vancomycin treatment failure. Therefore, selected MRSA strains from blood were further typed by MLST and PFGE in order to determine the molecular characteristics of MRSA bacteraemia in both years. In this study, the MRSA yield from blood for both years were accounted for 43.5% (*n* = 91) with 81.3% (*n* = 74) recorded in patients aged 51 years and more. Most of the bacteraemia cases were primary bacteraemia (*n* = 61; 67%). Diabetes (*n* = 32; 35.2%), hypertension (*n* = 35; 38.5%) and chronic kidney disease (*n* = 38; 41.8%) were the most common underlying comorbidities. MRSA bacteraemia were seen to be caused by HA-MRSA strains type III (*n* = 36) and IV (*n* = 15) and CA-MRSA strains type III (*n* = 15), IV (*n* = 9) and V (*n* = 2) as shown in Table 3. Analysis of the PFGE patterns have supported the possibility of the spread of SCC*mec* type III-ST239 into the community as they are closely related to the HA-MRSA clone of SCC*mec* type III-ST239. Whereas, SCC*mec* type IV strains were seen to have invaded and resided in this hospital as nosocomial MRSA strains. Due to their multiplication and transmission rates, there is a possibility for these type IV strains to replace and become the predominant endemic MRSA clone in this hospital [32].

Based on the PFGE dendrogram, 20 MRSA strains within Cluster I were closely related as they shared more than 80% similarity. Although they were from different years, they shared the same characteristics of having SCC*mec* type IV and ST22. MRSA strain of SCC*mec* type IV-ST1137 was also assigned in this cluster because ST1137 was shown to belong in the same CC as ST22. Another six SCC*mec* type IV MRSA strains were assigned in Cluster III as they shared similar characteristic of having MLST ST6. Most novel SCC*mec* type IV strains (*n* = 18) were assigned in the same Cluster I as SCC*mec* type IVb. SCC*mec* type IVa displayed no epidemiological linkage with SCC*mec* type IVb. There was a substantial genetic diversity in SCC*mec* type IV by the presence of various sequence types (ST1, ST5, ST6, ST22 and ST1137) indicating its capability to transfer between different genetic clones of *S. aureus.* All the MRSA strains that possessed SCC*mec* type III-ST239 were distributed in Cluster II.

The two SCC*mec* type V strains found in this study were CA-MRSA, PVL negative, had low vancomycin MIC and assigned as singleton due to difference in STs; ST772 and ST5. Previous studies done in the same hospital by Lim et al. [11] and Sam et al. [33] had reported the presence of ST22 and ST6 in SCC*mec* type IV and ST772 in SCC*mec* V. However, no local study has reported the presence of ST1 and S1137 in SCC*mec* type IV strains and ST5 in SCC*mec* type V strain. Interesting findings were that ST1-SCC*mec* IV has been reported by Otter and French in a study done on drug users and the homeless in South London [34] and ST5-SCC*mec* V was found in Japanese CA-MRSA isolates [35] which may indicate the invasion of these clones into Malaysia. The occurrence of the international major clones ST239-SCC*mec* III and ST22-SCC*mec* IV in Malaysia as well in other countries exhibited the epidemic nature of these clones [13]. To date, there is no study reporting ST1137-SCC*mec* type IV strains.

The untypeable SCC*mec* strains with ST239 and ST508 were not assigned to any clusters and appeared as singleton by PFGE. The untypeable CA-MRSA strain with novel ST508 might have derived from ST45 as it appeared to be a single locus (SLV) variant of ST45. According to Berglund et al. [36], MRSA strains that could not be typed by SCC*mec* typing might cause by some genetic rearrangement or mutations. This led to the emergence of a new highly pathogenic CA-MRSA clone which could have spread to the hospital. ST45 was previously reported as an epidemic strains in Germany and the Netherlands [37] and was present in MRSA bacteraemia isolates in a study done in Switzerland [38].

PFGE was shown to be more discriminatory than MLST in subtyping the MRSA strains. Based on PFGE, the strains had greater diversity with Simpson’s index of diversity, D of 0.946 (C.I.N.A. 0.916-0.976), on the other hand, for MLST, the strains were less diverse with Simpson’s index of diversity, D of 0.508 (C.I.N.A. 0.403-0.613). The majority of MRSA strains in this 2-year study period belonged to the pandemic clone SCC*mec* III-ST239 and most of them had pulsotype C. The predominance of this pulsotype could be due to the increase in nosocomial transmission within this hospital. In addition, the presence of this clone indicates the persistence of this clone and was identified as an endemic strain in this hospital [39].

### Limitations

The limitations for this study is the clinical data for some 2011 and 2012 MRSA strains could not be retrieved, and hence a comprehensive correlation between phenotypic, molecular and clinical data cannot be made.

## Conclusions

MRSA strains belonging to SCC*mec* type III were predominant in this hospital over the 2-year study period and the prevalence of PVL gene among 2011 MRSA strains were 5.3% with none detected in 2012 MRSA strains. The characteristics of MRSA strains that caused bacteraemia were genetically diverse by the presence of different clones circulating in this hospital, in which the majority belonged to SCC*mec* type III with MLST ST239 and exhibited pulsotype C. However, the presence of SCC*mec* type IV with MLST ST22 which is usually community-acquired is gaining prominence as they have become nosocomial pathogens and may replace the predominant SCC*mec* type III strains in this hospital. There were also emergence of novel clones which require further studies and proper monitoring as these clones may have epidemic and pathogenic potentials that could pose serious threat to public health.

## Abbreviations

CA: Community-acquired
CC: Clonal complex
CDC: Centers for Disease Control and Prevention
CLB: cell lysis buffer
CLSI: Clinical and Laboratory Standards Institute
CSB: Cell suspension buffer
CSF: Cerebrospinal fluid
FOX: Cefoxitin disk
HA: Hospital-acquired
MDROs: Multidrug resistant Organisms
MIC: Minimum inhibitory concentration
MLST: Multilocus sequence typing
MRSA: Methicillin-resistant *Staphylococcus aureus*
PCR: Polymerase chain reaction
PFGE: Pulsed-field gel electrophoresis
PVL: Panton-Valentine leukocidin
SCC*mec*: staphylococcal cassette chromosome *mec*
SSTIs: Skin and soft tissue infections
ST: Sequence type
TE: Tris-EDTA
TSA: Tryptic Soy Agar
UMMC: University Malaya Medical Centre
UPGMA: Unweighted pair group method with arithmetic averages
